# Data management issues for emerging diseases and new tools for managing surveillance and laboratory data.

**DOI:** 10.3201/eid0104.950403

**Published:** 1995

**Authors:** S M Martin, N H Bean

**Affiliations:** Centers for Disease Control and Prevention, Atlanta, Georgia, USA.


### Data Management Issues for Emerging Diseases and New Tools for Managing Surveillance and Laboratory Data

**Stanley M. Martin, M.S., Nancy H. Bean, Ph.D.**
Centers for Disease Control and Prevention, Atlanta, Georgia, USA

### Data Management Issues for Emerging Diseases

Since 1976, when Legionnaires' disease affected attendees at the American Legion Convention in Philadelphia (1), the scope of public health has expanded. During the 1976 outbreak investigation, public attention was drawn to news accounts of the increasing numbers of cases and deaths as well as to speculations about diseases causes and prevention. After the outbreak, public health officials contended with volumes of information, including clinical data, epidemiologic survey results, and records of specimens collected from patients and the environment. This information was managed on mainframe computers.

In 1980, a cluster of cases of unrecognized illness, primarily affecting young women, created a data management situation similar to that surrounding the Legionnaires' disease outbreak. A major epidemiologic investigation, which included examining a multitude of laboratory specimens and analyzing volumes of data, was undertaken by a large team of federal, state, and local public health officials, as well as numerous academic institutions and private industries. The problems with establishing databases and implementing a data management system for toxic shock syndrome (2) were essentially the same as the data management problems of Legionnaires' disease, except that computer technology had crept forward slightly in public health offices.

During the spring of 1993, a cluster of cases of another unknown illness, eventually attributed to hantavirus (3), occurred in the southwestern United States. The reaction to this unknown disease by public health officials reflected a startling fact: even though the epidemiologic and laboratory methods for curtailing the outbreak were in place, a consistent data management strategy had not been established. Ad hoc databases built by outbreak investigators for a multitude of purposes began to bog down the investigation. Cases were recorded in multiple databases that did not recognize duplicate reports of cases. Updates of data about cases were done in some, but not all, databases. Laboratory data about specimens from patients were not linked to other clinical and epidemiologic data about a patient. No single database was available with well-edited, complete data about all the cases. Parallel, fragmented data management efforts evolved in at least 15 locations, with no coordinated mechanism to integrate them into one system.

Introducing a single system for data management in the midst of the hantavirus outbreak involved more than the data management issues encountered in the earlier outbreaks. Previously, computer technology was viewed as a solution that, although somewhat cumbersome, enabled officials to move from data management by hand to electronic management. However, during the hantavirus outbreak, computer technology became part of the problem; it initially prevented good data management and may have hindered some of the laboratory and epidemiologic efforts to control the outbreak. Data were essentially being locked into various databases and could not be adequately analyzed or merged with data in other databases. In some instances, this peculiar circumstance caused investigators to perform analyses by hand using printouts from electronic databases or entering data again into other systems.

In recent years, legal considerations, such as the Privacy Act enacted in 1974 and the Freedom of Information Act enacted in 1966 (4,5), have also complicated data management. These acts, in their efforts to protect individual privacy and ensure availability of data, have in some cases, constrained public health responses to emergency situations and subsequent surveillance efforts by enforcing strict database design and handling requirements.

### Data Management Requirements

In epidemiologic investigations, disease problems are generally characterized by person, place, and time, whether the problem concerns the emergence of a new disease, a change in the resistance pattern of a known pathogen, an emergency response to an outbreak, or a routine disease surveillance program. The principles of data gathering, management, and analysis are essentially the same for all these purposes. Computer systems developed to manage data associated with these problems should be regarded as tools for the epidemiologic characterization of pathogens, syndromes, cases, and risk factors. Therefore, laboratory data management and reporting systems must be able to handle data about all of these.

The most stringent requirements for data management are imposed by data from laboratory testing of specimens from patients, human and nonhuman sources, and the environment. A system having a relational data model adequate to properly handle the laboratory data requirements will almost certainly be adequate to handle the clinical, exposure, and demographic data requirements.

Two primary data management functions can satisfy the laboratory data demands with multiple requirements in each function. The first function, internal laboratory data management, consists of entering test results and tracking specimens. The second, surveillance, includes gathering data and moving data beyond the electronic files of the laboratory to appropriate sites for analysis. A data management system should be able to perform these functions not only during an outbreak but throughout the period of surveillance as well.

The internal laboratory function, universally similar among most public health laboratories, includes data entry tailored for individual laboratories at the site; retrieval/query ability; and ability to add or delete tests, manage aliquots, share data input in different laboratories of the site, track the status of every specimen regardless of which laboratory tested it, develop reports for specimen submitters, and in some cases assign costs for laboratory tests performed and prepare invoices for submitters.

Requirements for the surveillance function include, in addition to certain critical laboratory data, the following facilities: to record clinical, exposure/risk factor, and demographic data about patients; to include data about multiple specimens and aliquots related to the same person, regardless of the interval separating the specimen dates; and to change questions or test results that are recorded for each specimen.

Although internal and surveillance functions are clearly separate, they are not independent. Data entered into databases for the internal function should be available without additional effort for the surveillance function. In fact, when the internal function is not electronic or when the internal electronic system is inadequate, the system performing electronic surveillance should also perform to some extent the internal functions. Good laboratory data management does not address the internal function at the exclusion of the surveillance function.

If a laboratory data management system is to be useful for emergency situations, it must provide mechanisms for adapting quickly to the emergency situation. For example, it must provide a way to immediately create an electronic data collection instrument and to incorporate this new instrument into the system at all reporting sites electronically. For the surveillance function, these electronic features must include communications facilities to move data electronically from one location to another, mechanisms for sending messages or files, functions for simple analysis, and methods for preparing and printing reports. While some systems perform some of these functions, most systems do not provide all of them.

With appropriate systems in hand, data management plans for both urgent and routine events can be approached in a sequential fashion. With consensus among all participating investigators, epidemiologists must decide what data (both laboratory and epidemiologic) are needed so that data field characteristics can be defined. Consensus should be reached in the early phase of the outbreak investigation; otherwise participants in the investigation will of necessity begin developing ad hoc data management systems. The more thoroughly and carefully this task is performed, the more stable the data will ultimately become.

In a well-designed system, the initial definitions in an emergency situation can include projections about which data fields will be needed. However, for routine surveillance these can be more thoroughly planned. Thus, the data system should allow fields to be deleted if not needed and to be added if they become important. These modifications should 1) be handled without having to alter the system, 2) use simple menu-driven functions requiring no computer programmer intervention, 3) accomplish the changes immediately, 4) be distributed to all investigators without disrupting their other functions during the investigation, and 5) be incorporated automatically.

Next, all known participants in the investigation must be identified. These should include local, state, and federal officials as well as academic or private participants who may provide reports to the central data repository. These participants must be identified to the system specifically by person and by site for system security. Appropriate state and federal offices should be informed concerning the computer system and the rules for its use well before an emergency occurs; therefore, sites will be on the system in advance of an urgent problem. However, the system must allow for additional sites to be added quickly. In an emergency, a temporary agreement must be drawn for all participants to cooperate with the demands of the situation, i.e., to use a particular software system and operate under a standard set of rules for collecting and reporting data for the emergency. This agreement may occasionally stipulate that participants share data temporarily in a common database for the sake of data integrity.

Entering clinical, epidemiologic/risk factor, and laboratory data about the same cases into the same database, rather than merging separate databases after the data are collected, provides such great payoffs in time savings and data integrity that the effort to obtain cooperation for a common database during an urgent situation is worthwhile. Although merging multiple databases during routine surveillance is feasible, emergency situations do not lend themselves to this type of data management. Therefore, the system to be used for these situations must accommodate a common database and provide a means of connecting the reporting sites to the database. When the reporting system is activated and data begin arriving at a central location, the system should facilitate analysis at every reporting site and provide a mechanism to export data (e.g., ASCII or .dbf files) for external analysis.

Emergency situations create unusual demands for epidemiologic and laboratory resources; therefore, data management should not disrupt or threaten to divert resources devoted to these other purposes. As the system is implemented, before emergencies occur, discussions of the resources required should be held with participants. Participants must devote some resources to data management, but these should be minimized. This is consistent with implementing a single system in the beginning of the outbreak investigation and continuing with it into the routine surveillance follow-up. Incorporating data into a second system for surveillance could waste resources.

Although, internal data management does not need to change to accommodate an outbreak, laboratories must implement systems that can directly feed data into the master reporting system database, either through an import function contained in the master system or by a direct interface between the internal laboratory system and the surveillance reporting system.

Data management considerations during outbreak investigations and surveillance in the United States include the political concerns of the participants. Political and legal constraints of all participants must be addressed before the need to deal with them arises. On a global scale, this consideration is equally important, especially in countries whose economies may be adversely affected by news of a dangerous disease situation. Individual country sovereignty must not be violated by data reporting, and the cooperation of each participating country or political entity (e.g., World Health Organization [WHO], Pan American Health Organization [PAHO]) must be obtained in an atmosphere of confidentiality. All attempts to obtain, share, or combine data on a regional or global basis must include a well-defined set of rules agreed upon by all participants. For example, data for scientific purposes might be received at an office of WHO or PAHO but not sent beyond these organizations.

Most often, for the sake of surveillance on a regional or global scale, data management considerations must focus first on establishing in-country data management infrastructures. This means that regional or global surveillance will first translate into establishing a master system, or at least compatible systems in individual participating countries. In most cases, data management systems available to developing countries do not provide the relational model needed by the laboratory. Therefore, efforts should be initiated to introduce and establish systems that can meet these needs in countries desiring to use them.

A plan for regional or global surveillance must include tools to respond to outbreaks and provide for the computing equipment and modems or other means of transmitting the data electronically. Today's environment demands that most data management be done on personal computers located at critical sites where data can be input. However, data volume may ultimately require that the system provide for archiving data onto another medium. This does not preclude the use of personal computers for data management but simply recognizes that current technology limits the volume of data that can practically be managed and analyzed on personal computers.

The initial data management plan for a country should include a section on reporting procedures and the appropriate medium for archiving data. To handle an immediate, urgent situation the system should contain, at a minimum, a personal computer with large hard-disk capacity (at least 1-2 gigabytes at the central level and possibly 300-500 megabytes at each reporting site), large memory (at least 4 megabytes of RAM at every reporting site), adequate speed (at least 33 megahertz at every reporting site), and fast modems if appropriate. For sites located in areas with inadequate telephone lines, other provisions for electronic transmissions should be planned (e.g., diskettes). Until security can be assured on the Internet, we do not recommend using this medium for electronic transmission of laboratory clinical data for outbreak investigations and surveillance.

### New Tools for the Management of Surveillance and Laboratory Data

### The Public Health Laboratory Information System (PHLIS)

To address the need for a data management system for outbreak investigations and surveillance, the National Center for Infectious Diseases, CDC, in cooperation with the Association of State and Territorial Public Health Laboratory Directors in the United States, developed PHLIS. With this system, data entry screens (modules) are created and distributed to all reporting sites electronically, and data are input and reported within hours, without involving computer programmers. PHLIS provides the capacity for a hierarchical reporting scheme involving reports to multiple, successively higher reporting levels; a database is created at every reporting level so that all data reported to a site or input at the site are included in the database at that site.

The most recent version of PHLIS (Version 3.0), is a menu-driven system based on a relational data model sufficient for the needs outlined in the first part of this report. The system allows for a patient record to be input only one time and links multiple specimens for that patient record. This is true even if specimens for the same patient are entered in different disease modules, or if the patient's name is to be added into a module that contains only epidemiologic data (no laboratory specimens). PHLIS provides a core set of data to be collected on every patient. In addition, each disease module can be customized by adding additional fields to the core data if needed. The system can accommodate data for epidemiologic, laboratory, survey, and case-control studies, and for other public health needs.

Field staff can rapidly add their own data fields to existing disease modules to customize the data entry for special needs at each data reporting site. During an outbreak, a new module can be rapidly developed and electronically transmitted to all participating reporting sites.

The system, which includes data communication software, is configured so that data flow in a pyramid reporting structure: that is, data are reported from lower level reporting sites through higher level reporting sites and ultimately to a single central site. As data are passed to each successively higher level, they are automatically assimilated into that site's database. Thus, databases are built and updated at successively higher reporting sites. Additional information about a case or specimen may be added at any reporting site; if desired, these additional data are also transmitted to the next higher reporting site.

To meet the need for feedback, PHLIS has a menu-driven option to transmit files or messages up and down the reporting chain, with these files and messages being transmitted automatically when connections are established for each data transmission. This facility is flexible enough to allow any valid user in the reporting chain to transmit files or messages to any other user in the reporting chain. For example, in the United States, a county health official who is included in the reporting system in one state can send messages or files to a participating county official in another state. The feedback system does not mimic electronic mail because these files and messages are sent along the reporting chain in the same communications configuration as data reporting. Therefore, successful arrival of these messages at their destination(s) depends upon each member of the reporting chain between the sender and the receiver to establish a connection for reporting purposes. However, the system provides an alternative mechanism for sending files and messages directly to any other reporter having the capacity to receive them without going through the reporting chain.

PHLIS is used in all 50 state public health laboratories, as well as the District of Columbia and Guam. Disease modules included are animal rabies, *Campylobacter, Escherichia coli* O157:H7, Lyme disease, mycobacteria, respiratory and enteric viruses, human *Salmonella*, nonhuman *Salmonella*, *Shigella*, and drug-resistant *Streptococcus pneumoniae.*

PHLIS can be implemented independently: organizations can develop their own PHLIS pyramid reporting system. For example, PHLIS is currently being implemented at the Caribbean Epidemiology Center (CAREC) in Trinidad and in its member countries for the reporting of HIV/STD infections with the expectation that the reporting system will be expanded to accommodate other diseases. CAREC can receive reports from the member countries as each country is added to the reporting structure.

### Laboratory Information Tracking System (LITS)

The second system, LITS, is a PC local area network-based system for tracking laboratory specimens. The system allows specimen information to be entered at a central specimen receiving site; additional information about the specimen can be entered into the system in any of the laboratories performing tests on that specimen. Although modules are customized for each laboratory's needs, laboratorians can add additional tests or delete obsolete ones. Furthermore, users can examine all the data about a specimen, including data from all laboratories that performed tests on the specimen. Other features in the system include cost billing, user defined reports, user defined query, and specimen or patient tracking and security. For emerging diseases, LITS provides a mechanism to standardize laboratory protocol across organizations and a mechanism to share data about specimens within an organization.

### Acknowledgments

We thank Tim Kuhn for leading the programming team; Bruce Wilson, Dana Crenshaw, Joe Bates, and Neil Jones for programming support; Tim Day for user support; Kathleen Maloney, Joy Goulding, Lori Hutwagner, and Cecile Ivey for evaluating program integrity; Brian Plikaytis for his early involvement with LITS; and Cheryl Shapiro for financial management.

### References

1. Fraser DW, Tsai TR, Orenstein W, Parkin WE, Beecham HJ, Sharrar RG. Legionnaires' disease: description of an epidemic of pneumonia. N Engl J Med 1977;297:1189-97.
2. Shands KN, Schlech WF III, Hargrett NT, Dan BB, Schmid GP, Bennett JV. Toxic shock syndrome: case-control studies at the Centers for Disease Control. Ann Intern Med 1982;96:895-8.
3. CDC. Outbreak of acute illness—southwestern United States, 1993. MMWR 1993;42:421-4.
4. Administrative Conference of the U.S. Privacy Act. In: Federal Administrative Procedure Sourcebook, 2nd ed. Office of the Chairman, 1992:863-979.
5. Administrative Conference of the U.S. Freedom of Information Act. In: Federal Administrative Procedure Sourcebook, 2nd ed. Office of the Chairman, 1992:633-61.
